# Intraocular Pressure Elevation Compromises Retinal Ganglion Cell Light Adaptation

**DOI:** 10.1167/iovs.61.12.15

**Published:** 2020-10-16

**Authors:** Xiaofeng Tao, Jasdeep Sabharwal, Samuel M. Wu, Benjamin J. Frankfort

**Affiliations:** 1 Department of Ophthalmology, Baylor College of Medicine, Houston, Texas, United States; 2 Department of Neuroscience, Baylor College of Medicine, Houston, Texas, United States

**Keywords:** ganglion cell, RGC, glaucoma, intraocular pressure, light/dark adaptation

## Abstract

**Purpose:**

Functional adaptation to ambient light is a key characteristic of retinal ganglion cells (RGCs), but little is known about how adaptation is affected by factors that are harmful to RGC health. We explored adaptation-induced changes to RGC physiology when exposed to increased intraocular pressure (IOP), a major risk factor for glaucoma.

**Methods:**

Wild-type mice of both sexes were subjected to 2 weeks of IOP elevation using the bead model. Retinas were assessed using a multielectrode array to record RGC responses to checkerboard white noise stimulation under both scotopic and photopic light levels. This information was used to calculate a spike-triggered average (STA) for each RGC with which to compare between lighting levels.

**Results:**

Low but not high IOP elevation resulted in several distinct RGC functional changes: (1) diminished adaptation-dependent receptive field (RF) center-surround interactions; (2) increased likelihood of a scotopic STA; and (3) increased spontaneous firing rate. Center RF size change with lighting level varied among RGCs, and both the center and surround STA peak times were consistently increased under scotopic illumination, although none of these properties were impacted by IOP level.

**Conclusions:**

These findings provide novel evidence that RGCs exhibit reduced light-dependent adaptation and increased excitability when IOP is elevated to low but not high levels. These results may reveal functional changes that occur early in glaucoma, which can potentially be used to identify patients with glaucoma at earlier stages when intervention is most beneficial.

Glaucoma is degenerative disease of retinal ganglion cells (RGCs) and the optic nerve. Traditionally, significant RGC and optic nerve injury was thought to be required before measurable visual dysfunction occurred,^1^^,^^2^ but there is also evidence for diffuse losses in contrast sensitivity in early glaucoma^3^.^3^ Many patients with glaucoma endorse visual complaints even when acuity is good, especially under dim lighting conditions, suggesting functional decline.^4^^–^^6^ Contrast sensitivity testing suggests that glaucoma patients may not adapt to changes in lighting condition normally.^7^^,^^8^

Intraocular pressure (IOP) is the only known modifiable risk factor for glaucoma, and is associated with both disease presence and progression.^9^^,^^10^ To study the effects of IOP on RGC and optic nerve anatomy and function, mouse models of IOP elevation have been of great value.^11^^–^^14^ Recent studies show that mouse RGCs that are exposed to elevated IOP are rapidly dysfunctional, long before cell death occurs.^12^^,^^15^^–^^19^ Interestingly, several studies have also identified nonlinear effects of IOP elevation on RGC function prior to RGC death, including threshold effects,^18^ opposing effects based on short- or long-term IOP exposure,^20^ and opposing transcriptional effects of low and high IOP elevation.^21^

RGCs are the obligate output neuron of the retina and successfully integrate both rod (scotopic) and cone (photopic) dominated signals. Accordingly, functional adaptation to changes in light level is a core property of RGCs.^22^^–^^29^ However, the effects of IOP on RGC light-dependent adaptation are unknown. This manuscript extends our understanding of RGC light-dependent adaptation and reports novel effects of IOP elevation on this property. Using multielectrode array recordings to interrogate the properties of individual RGCs following IOP elevation to specific ranges, we find that light-dependent RGC adaptation is impaired by low but not high elevation of IOP. This suggests that abnormal RGC light adaptation is an early effect of IOP elevation, which may later be undetectable due to additional RGC dysfunction at higher IOP levels or longer durations of IOP exposure. This finding may offer a new opportunity for the detection of glaucoma in the early stages of human disease, prior to permanent visual dysfunction.

## Methods

### Experimental Animals

Twelve-week old C57BL6J mice of both sexes were purchased from Jackson Laboratory (stock no. 000664). Mice were kept at Baylor College of Medicine according to a standard 12/12 light cycle. All animal care was approved by the Institutional Animal Care and Use Committee of Baylor College of Medicine in accordance with the ARVO Statement for the Use of Animals in Ophthalmic and Vision Research.

### Mouse Model of IOP Elevation

**Figure 1.**
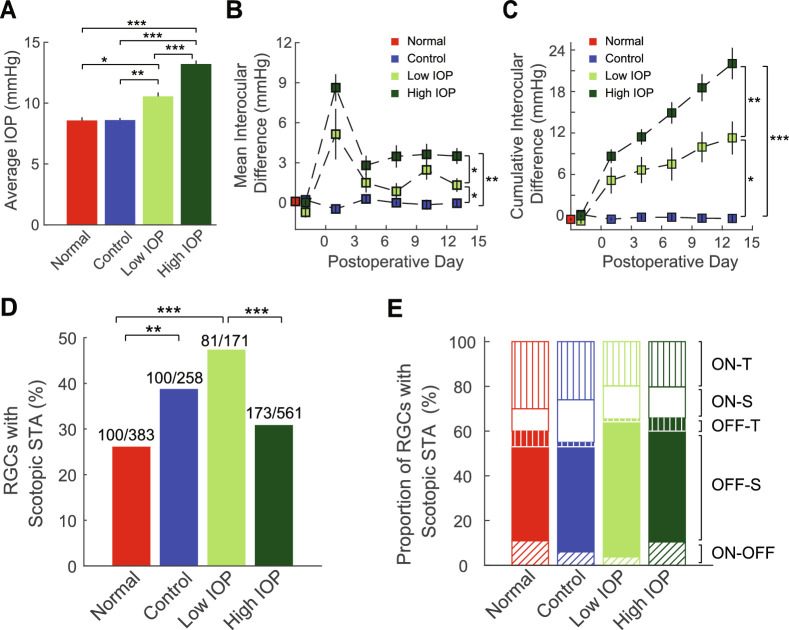
IOP and RGC sampling. For all panels, *red* = untreated eyes (normal), *blue* = saline injected eyes (control), *bright green* = bead injected eyes (low IOP), and *dark green* = bead injected eyes (high IOP). (**A**) Average IOP throughout the 2-week study. (**B**) Average interocular IOP difference per day throughout the 2-week postoperative period. *Vertical bars* represent 1 SEM. (**C**) Cumulative interocular IOP difference per day throughout the 2-week postoperative period. *Vertical bars* represent 1 SEM. (**D**) Among sampled RGCs, the proportion of the cells that showed a scotopic STA. Numbers above each bar indicate how many RGCs showed photopic STAs (denominator) and scotopic STAs (numerator). (**E**) Among RGCs that showed scotopic STAs, the proportion of ON-transient cells (ON-T), ON-sustained cells (ON-S), OFF-transient cells (OFF-T), OFF-sustained cells (OFF-S), or ON-OFF cells for each group. Open areas = ON cells, filled areas = OFF cells, vertical lines = ON or OFF transient cells; no lines = ON of OFF sustained cells, and oblique lines = ON-OFF cells. There is no difference in the distribution of RGC subtypes according to experimental group. *P < 0.05; **P < 0.01; ***P < 0.001. *Vertical lines* indicate 1 SEM.

Polystyrene bead anterior chamber injection was utilized to produce IOP elevation and has been previously described.^12^ Animals were initially anesthetized by intraperitoneal injection of a combination anesthetic (ketamine 37.5 mg/mL, xylazine 1.9 mg/mL, acepromazine 0.37 mg/mL). Topical anesthesia was added by administrating 0.5% proparacaine ophthalmic solution to the cornea. Once adequate anesthesia was confirmed, 1.5 µL of beads followed by 3.0 µL of viscoelastic (sodium hyaluronate) were injected into the anterior chamber of the left eye. In the control group, the beads were replaced by the same volume of sterile saline. In the normal group, no injection was performed. Following injection, animals were monitored for up to 15 days. IOPs of both injected eyes and normal contralateral eyes (uninjected, right eyes) were measured with a rebound tonometer (Icare Finland OY, Vantaa, Finland)^30^ three times a week, at intervals of no more than 3 days. Each recorded IOP value was an average of six measurements. To minimize the effect of diurnal fluctuation, IOP was measured within a controlled time window (10 AM to 2 PM). IOP was then assessed as both an average of all measurement points postinjection (Fig. 1A) and as an average or cumulative IOP difference (injected eye – uninjected eye) over time (Figs. 1B, 1C). One eye from 48 animals were used in the study: 26 eyes injected with beads (IOP), 7 eyes injected with saline (control), and 15 eyes with no injection (normal).

Animals with elevated IOP were stratified into low (*n* = 7) and high (*n* = 19) groups according to average IOP elevation. Elevation of 1 to 4 mm Hg defined the low IOP group, and IOP elevation greater than 4 mm Hg defined the high IOP group.

### Multielectrode Array (MEA) Recording

MEA recording and measurements of RGC physiology parameters were conducted 15 days following bead or saline injection. MEA procedures were as previously described.^17^^,^^18^^,^^31^^,^^32^ All animals were dark-adapted for a minimum of 2 hours before euthanasia for electrophysiological recording. Retinas were dissected (including removal of retinal pigment epithelium) in a carboxygenated buffer (NaCl 124 mM, KCl 2.5 mM, CaCl_2_ 2 mM, MgCl_2_ 2 mM, NaH_2_PO_4_ 1.25 mM, NaHCO_3_ 26 mM, glucose 22 mM, pH titrated to 7.35, bubbled with 95% O_2_ and 5% CO_2_) under infrared illumination (BE Meyers & Co., Inc., Redmond, WA, USA). Each dissected retina was flat-mounted on a black, gridded microfilter membrane (EMD Millipore, Burlington, MA, USA) and transferred to the array (MEA-60; Multichannel System MCS GmbH, Reutlingen, BW, Germany) with its inner surface (RGC layer) in contact with the electrodes. The retina was perfused with the dissection buffer and kept at 36.5°C throughout the entire experiment. Spiking signals of the RGCs were collected from 60 electrodes that were arranged in an 8 × 8 grid covering an area measuring approximately 0.6 mm^2^. RGC signals were sampled at 20 KHz before passing through a 0.1 Hz high-pass filter. Spike isolation and sorting procedures were executed offline in MATLAB (MathWorks Inc., Natick, MA, USA).

### Light Level Calibration

Visual stimuli were generated with PsychToolbox (MATLAB), displayed on an OLED microdisplay (eMagin Inc., Hopewell Junction, NY, USA), and optically projected to the retina via a beam splitter (Edmund Optics Inc., Barrington, NJ, USA). Ambient light level for photopic stimulation was calibrated to 3.07 log_10_(R*/rod/sec; S170C power sensor from ThorLabs Inc., Newton, NJ, USA and SpectraRad spectrometer from B&W Tek, Newark, DE, USA) at the RGC plane. Scotopic ambient light was achieved by adding neutral density filters in the light pathway and was measured to be –0.93 log10(R*/rod/sec). Calibration was conducted prior to all experiments. Each experiment started with scotopic stimulation and was followed by photopic stimulation.

### Spontaneous Firing

Spontaneous firing of RGCs was recorded during scotopic experiments. Spikes were collected when the retina was not visually stimulated and was only exposed to ambient light.

### Light Response Polarity

Photopic whole-field dark or bright stimulation was used to determine the polarity of light response of the RGCs. Alternating uniform black and white screens were presented every 4 seconds, and this black-white cycle was repeated for twelve trials. RGC preference to brightness or darkness was determined by computing an ON-OFF index according to Equation 1.

(1) $$\begin{array}{c} \;\;\begin{array}{c} ON\;-\;OFF\;Index\;=\;\left(Response_{ON}\;-\;Response_{OFF}\right)/\left(Response_{ON}\;+\;Response_{OFF}\right), \end{array}\;\; \end{array}$$

where *Response_ON_* and *Response_OFF_* are the spike counts when screen was white or black. ON cells had an ON-OFF index greater than 0.7, and OFF cells had an index less than –0.7. All other cells were classified as ON-OFF cells. This ON-OFF classification was further verified qualitatively by visually inspecting each RGC's responses.

### Center and Surround Receptive Field (RF)

Dynamic white noise checkerboards were used to determine RGC RFs. Each element of a checkerboard was a white or black square (50 µm side) flickering at 15 Hz. Each checkerboard had 32 × 32 squares. A set of checkerboards (20,625) were created in PsychToolbox^33^^,^^34^ and presented to the retina continuously.^17^^,^^32^^,^^35^ The presentation time for the whole set was approximately 90 minutes for each light level (scotopic or photopic). During this period, RGC spikes were collected and saved for offline analysis, in which spikes were reverse-correlated to the checkerboard frames for the calculation of space-time spike-triggered averages (STAs).^36^^–^^38^ These STAs were fit to the product of a two-dimensional spatial Gaussian and the impulse response of a temporal filter.^36^ The quality of STA fitting was assessed by r^2^ that was calculated for the entire 32 × 32 space-time map. Spatial and temporal characteristics of an RF were evaluated once the fitting was reasonably good (r^2^ for the entire map should be >0.3).

The spatial Gaussian of the STA was used to determine RGC RF size (Equation 2):

(2) $$RF\;size=sqrt\left(\sigma_{x}*\sigma_{y}\right),$$

where σ_x_ and σ_y_ represent 1-σ distance in major and minor axis, respectively. This represents the spatial boundary (1σ) of the center RF^17^^,^^18^^,^^32^^,^^35^ (Supplementary Fig. S1). Using these inclusion criteria, we identified 383 normal RGCs, 258 control RGCs, 171 low IOP RGCs, and 561 high IOP RGCs. The number of RGCs collected per retina was equivalent across all experimental conditions (ANOVA; *P* > 0.05).

To study the spatial interaction between the center and surround RF, an area that expands to nine times of its spatial extension (9σ) was subjected to extended computational analysis (see Fig. 4A). First, this area was separated by an annulus to define the center RF and the surround RF. The integral of the temporal impulse functions derived from the center RF and surround RF were calculated separately, and the ratio of the surround integral to the center integral was defined as the relative surround strength (RSS; see Fig. 4A). While maintaining the surround area constant at 9σ, we then increased the center annulus from 1σ to 8σ in one σ steps and recalculated the RSS for each annulus size. This generated an RSS-to-σ function for each RGC. The *x*-axis position of the lowest point of this function indicates the spatial position (in terms of σ), where the transition from center RF to surround RF most likely occurs. We termed this point the “transitional zone” (see Fig. 4B). The spatial shift of the transitional zone under different light levels characterizes the light-dependent, spatial interaction between the center and surround RFs. We then compared the shift of the transitional zone qualitatively and quantitatively for normal, control, and IOP elevated RGCs.

Temporal characteristics of the RF were studied by quantifying STA peak time for both center (1σ to 3σ) and surround (4σ to 9σ) areas. This separation of center and surround was empirical and allowed us to compare our results with previously published studies.^17^^,^^32^^,^^35^

### Experimental Design and Statistical Analysis

Data are presented in the figures with means and SEMs. ANOVAs were used to test the significance (α = 0.05) of the differences between groups. When ANOVA results were significant and warranted comparisons between groups, *P* values were adjusted for multiple comparisons using Fisher's Least Significant Difference procedure.

## Results

### IOP Elevation and RGC Sampling

In this study, we included normal eyes (no procedure), experimental eyes (elevated IOP), and control eyes (procedure without elevated IOP). We elevated IOP in experimental eyes with the bead + sodium hyaluronate injection model (control eyes = saline + sodium hyaluronate) as previously described.^11^^,^^12^ In normal eyes, the average IOP was 8.57 ± 0.34 mm Hg (mean ± SEM). In control eyes, the average IOP was equivalent (8.64 ± 0.23 mm Hg). In experimental eyes, the IOP increased to various degrees. We therefore stratified experimental eyes according to the magnitude of IOP increase to identify low and high IOP groups (see Methods) because these IOP levels appear to define unique physiologic and transcriptional states.^18^^,^^21^ Overall, in the low IOP group the average IOP was 10.66 ± 0.47 mm Hg (average increase of 2.09 mm Hg) and in the high IOP group the average IOP was 13.25 ± 0.40 mmHg (average increase of 4.67 mm Hg). The average IOP of the high IOP group was higher than that of the low IOP group, and both were significantly higher than normal or control eyes (Fig. 1A). These distinctions were maintained when IOP values were plotted as the average and cumulative IOP differences (IOP of injected eye – IOP of uninjected eye) over time (Figs. 1B, 1C).

Next, we identified RGCs using an established MEA technique based on the calculation of an STA of responses to a rapidly flickering white noise checkerboard under both scotopic and photopic conditions.^17^^,^^32^^,^^35^ Because nearly all RGCs have a detectable photopic STA with this approach, we selected RGCs based on the presence of a photopic STA.^35^ We identified 383 normal RGCs, 258 control RGCs, 171 low IOP RGCs, and 561 high IOP RGCs (Methods). To rule out sampling bias, we checked RGC density (the number of recorded RGCs per retina) for each treatment group and found that there was no difference in RGC yield per retina among the 4 groups (ANOVA, *P* = 0.43). Consistent with previous results, not all RGCs recorded a scotopic STA.^17^^,^^35^ Interestingly, the proportion of total RGCs that showed both photopic and scotopic STAs was not uniform across IOP groups (Fig. 1D). In normal eyes, 26.1% of RGCs had an identifiable STA in both lighting conditions. In control eyes, 38.8% of RGCs had an identifiable STA in both lighting conditions, a 49% increase from normal eyes (*P* < 0.01; χ^2^ test). In the low IOP group, 47.4% of RGCs had an identifiable STA in both lighting conditions, an 82% increase from normal eyes (*P* < 0.001; χ^2^ test). In the high IOP group, 30.8% of RGCs had an identifiable STA in both light conditions, which was unchanged from normal eyes.

To examine the specific effects of elevated IOP on light-dependent RGC physiology (i.e., scotopic to photopic transition), we therefore further analyzed only RGCs with measurable photopic and scotopic STAs (100 normal, 100 saline, 81 low IOP, 173 high IOP) for the rest of this manuscript. Consistent with previous results, among RGCs with both photopic and scotopic STAs, there was no difference in the distribution of RGC subtypes (Fig. 1E; χ^2^ test, *P* = 0.07). The percentages of ON, OFF, and ON-OFF cells in this population were similar to those in published studies.^15^^,^^39^^,^^40^ Because there were very few OFF-transient type RGCs with this further subdivision, we pooled sustained and transient cells together according to ON or OFF properties. With this pooled classification method, there was again no difference in RGC type distribution between treatment groups (χ^2^ test, *P* = 0.19), and therefore it was used for the remainder of the study.

**Figure 2.**
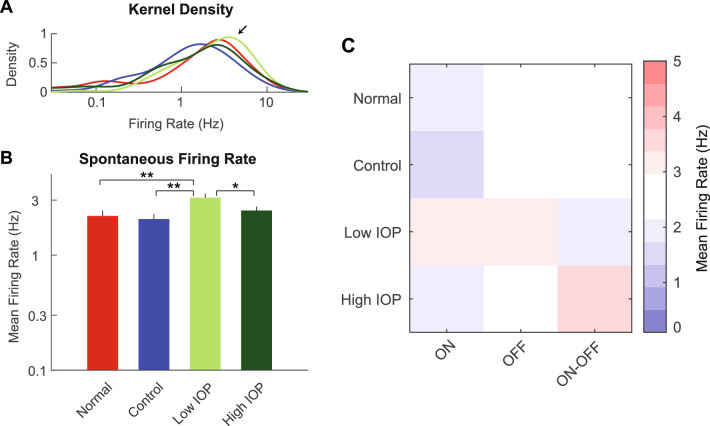
RGC spontaneous firing rates. (**A**) Kernel density estimation of firing rate distribution for normal (*red*), control (*blue*), low IOP (*bright green*), and high IOP (*dark green*) groups. *Arrow* indicates a right shift of the distribution in low IOP group toward higher spontaneous firing rates. (**B**) Average spontaneous firing rate compared among the four groups. The spontaneous firing rate is increased in the low IOP group only. (**C**) Heatmap shows the average spontaneous firing rate for each RGC subtype according to experimental group. *P < 0.05; **P < 0.01. *Vertical lines* indicate 1 SEM.

First, we determined the spontaneous firing rate for RGCs with both photopic and scotopic STAs (Fig. 2). The spontaneous firing rate of RGCs in the low IOP group was shifted toward higher rates (Fig. 2A). Overall, the average spontaneous firing rate was 2.18 Hz for normal RGCs and 2.05Hz for control RGCs, confirming that the injection procedure did not impact basal RGC activity (Fig 2B). However, RGC spontaneous firing rate was increased to 3.15 Hz in the low IOP group (*P* < 0.01 to both normal and control eyes). In the high IOP groups, this increase in spontaneous firing rate diminished from the low IOP group (*P* < 0.05 between low and high IOP) back to the range of the normal and control groups at 2.43 Hz. This result suggests an increased excitability of RGCs after IOP elevation, but only in the low IOP group. Further analysis of ON, OFF, and ON-OFF cells showed that ON cells may have lower firing rates in general across all study populations (Fig. 2C and Supplementary Table S1; *P* = 0.0072) but there were no IOP-dependent subtype-specific effects (*P* = 0.62; Supplementary Table S1).

### Spatial Characteristics of the Center and Surround RFS

**Figure 3.**
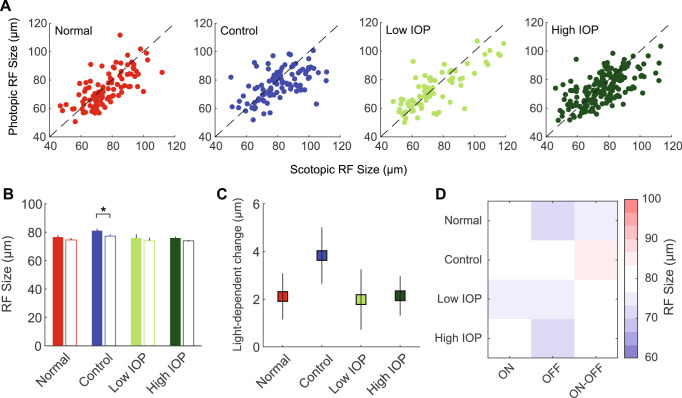
Scotopic and photopic RF center size. (**A**) Scatter plot of the scotopic (*x*-axis) and photopic (*y*-axis) RF center size for RGCs in normal (*red*), control (*blue*), low IOP (*bright green*), and high IOP (*dark green*) groups. (**B**) Average RF center size for each group. *Filled bars* represent scotopic RF center size and *empty bars* represent photopic RF center size. (**C**) Changes in RF size due to light level change (scotopic -- photopic). (**D**) Heatmap shows the average RF size for each RGC subtype according to experimental group. *P < 0.05. *Vertical lines* indicate 1 SEM.

We next explored the spatial characteristics of the scotopic and photopic RFs, with specific attention to light-dependent changes in RF properties. First, we plotted the RF center size for each RGC and compared diameters between scotopic and photopic lighting conditions. We found a wide range of RF center size changes in both directions with movement from scotopic to photopic light (Fig. 3A). On average, scotopic and photopic RF center size diameters were the same, except for control RGCs (Fig. 3B). When compared as an average light-dependent change in RF center size, there were no differences among experimental groups (Fig. 3C). Analysis on subtypes showed that ON cells as a whole group may have relatively larger RF size (Fig. 3D and Supplementary Table S1; *P* = 0.0033) but there were no IOP-dependent subtype specific effects (*P* = 0.33; Supplementary Table S1).

**Figure 4.**
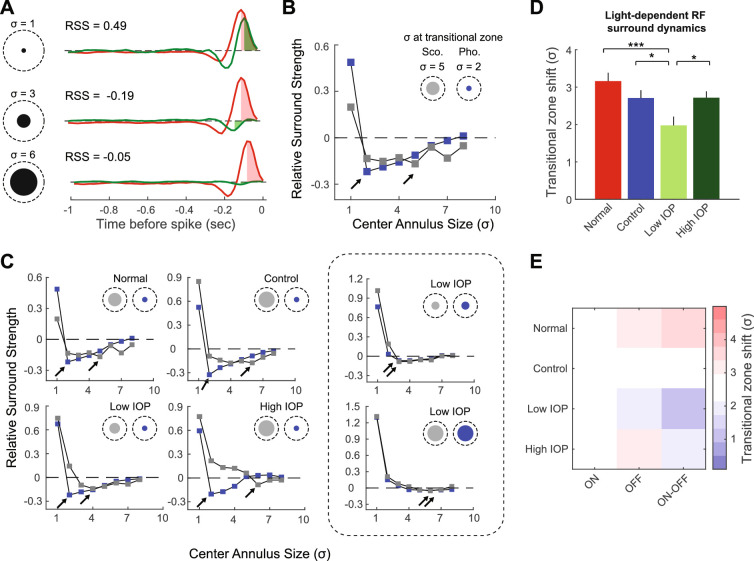
Light-dependent changes in RSS are modified by low IOP. (**A**) Calculation of RSS according to center annulus size for a normal RGC. A center annulus is used to define the center RF and is varied according the size of the STA (σ, *black circles*, examples of 1, 3, and 6 σ are shown). The maximum extent of the spatial RF (9σ; *dashed circles*) is fixed. For each annulus definition, the temporal impulse functions for the center RF (*red trace*) and surround RF (*green trace*) were obtained and plotted. *Red* and *green shaded* areas are the integrals (from spike time to peak) for center RF and surround RF, respectively. The RSS was calculated as the ratio of the surround integral to the center integral (shown for each example, see Methods). Examples of RSS values for three different center annulus sizes (σ = 1, 3, or 6). Negative RSS values indicate opposite polarity of the surround. (**B**) For the same RGC in (A), the RSS was calculated with center annulus size increases from 1σ to 8σ, in one σ steps, and plotted as a function of σ. This was done for both the scotopic (*gray*) and the photopic (*blue*) STAs. The lowest point of each function indicates a transition from center to surround and is called the “transitional zone” (*arrows*). *Circles* at the upper right corner depict the area within the transitional zone under scotopic (*gray*) or photopic (*blue*) light. Note that the transitional zone shifts significantly (3σ distance) when light level changes. (**C**) RSS plots for the same normal RGC as in panel (B) and five additional example RGCs from control, low IOP, and high IOP groups. The *dashed rectangle* highlights two example RGCs from the low IOP group that showed loss of spatial shift in the transitional zone. (**D**) Average spatial shift of the transitional zone due to light level changes in normal (*red*), control (*blue*), low IOP (*bright green*), and high IOP (*dark green*) groups. (**E**) Heatmap shows the average transitional zone shift (σ) for each RGC subtype according to experimental group. *P < 0.05; ***P < 0.001.

Second, we evaluated the RF center-surround interaction by calculating the RSS for each RGC and comparing light-dependent spatial dynamics among experimental groups (Fig. 4A; see Methods). RSS, a ratio of temporal impulse function integrals between surround and central RFs, is a measurement of relative strength of the surround. RSS was used to determine the location of the transitional zone (the position where the minimum RSS was found). In normal, control, and high IOP groups, RGCs displayed robust light-dependent dynamics in transitional zones (Figs. 4B, 4C). In these cells, the transitional zone showed a significant spatial shift with change from scotopic to photopic conditions. This shift typically included increased surround area with concomitant reduction in the size of the center RF (Fig. 4B inset). Although this pattern was also found in some of the RGCs from the low IOP group, we noticed that a large proportion of RGCs in the low IOP group showed no such light-dependent transitional zone shift (Fig. 4C and Table; χ^2^ test, *P* = 0.014). We quantitatively assessed this spatial shift for all groups and found that the average transitional zone shift in the low IOP group was significantly smaller than any other group (Fig. 4D; *P* = 0.0003 compared with normal, *P* = 0.025 compared with control, and *P* = 0.012 compared with high IOP). Thus RGCs in the low IOP group, but not the high IOP group, showed impaired light-dependent center-surround dynamics. Analysis of RGC subtypes (Fig. 4E) confirmed a significant IOP effect (Supplementary Table S1; *P* = 0.022) but there were no IOP-dependent subtype specific effects (*P* = 0.58).

**Table.** RGCs Showing a Shift in Transitional Zone

	Total	TZ Shift		No TZ Shift			
	*n*	*n*	%	*n*	%	χ^2^	*P*
**Normal**	100	73	73%	27	27%		
**Control**	100	71	71%	29	29%		
**Low IOP**	81	42	52%	39	48%		
**High IOP**	173	116	67%	57	33%		
**Statistics**						10.63	0.0139

TZ, transitional zone.

### Temporal Characteristics of the Center and Surround RFS

**Figure 5.**
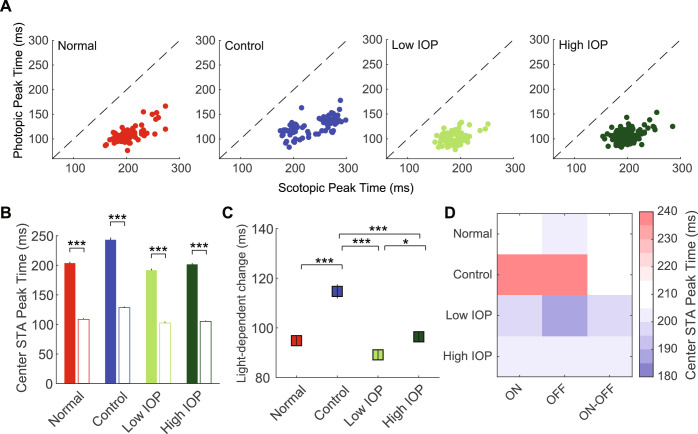
Scotopic and photopic center STA peak time. (**A**) Scatter plot of the scotopic (*x*-axis) and photopic (*y*-axis) center STA peak time for RGCs in normal (*red*), control (*blue*), low IOP (*bright green*), and high IOP (*dark green*) groups. (**B**) Average center STA peak time for each group. *Filled bars* represent scotopic peak time; *empty bars* represent photopic peak time. (**C**) Changes in peak time due to light level change (scotopic -- photopic). (**D**) Heatmap shows the average center STA peak time for each RGC subtype according to experimental group. *P < 0.05; ***P < 0.001. *Vertical lines* indicate 1 SEM.

**Figure 6.**
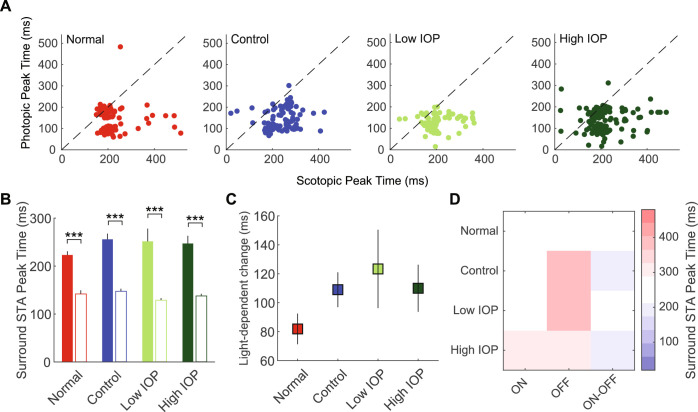
Scotopic and photopic surround STA peak time. (**A**) Scatter plot of the scotopic (*x*-axis) and photopic (*y*-axis) surround STA peak time for RGCs in normal (*red*), control (*blue*), low IOP (*bright green*), and high IOP (*dark green*) groups. (**B**) Average peak time for each group. *Filled bars* represent scotopic peak time; *empty bars* represent photopic peak time. (**C**) Changes in peak time due to light level change (scotopic -- photopic). (**D**) Heatmap shows the average surround STA peak time for each RGC subtype according to experimental group. ***P < 0.001. *Vertical lines* indicate 1 SEM.

Finally, we explored the temporal characteristics of the scotopic and photopic RFs, again with specific attention to light-dependent changes in RF properties. As expected, in all groups, the center STA (defined as 1-3σ of the Gaussian; see Methods) peak time was significantly increased in scotopic compared with photopic light (Figs. 5A, 5B). When compared among experimental groups as an average light-dependent change in center STA peak time, control RGCs had a significantly larger increase under scotopic lighting when compared with all other groups (Fig. 5C). This IOP-dependent effect was present across RGC subtypes (Fig. 5D and Supplementary Table S1; *P* < 0.001). However, it diminished in RGCs exposed to any IOP elevation, more so among those in the low IOP group than the high IOP group. Together, this suggests a compensation of the space-time center RF integration in control eyes, likely induced by the experimental procedure, with a secondary effect of IOP elevation, consistent with previous studies.^18^ When we looked at the temporal properties of the RF surround (defined as 4-9σ of the Gaussian), as expected, under scotopic lighting most RGCs showed an increased peak time, regardless of experimental group (Fig. 6A), which yielded an increased average peak time for all groups (Fig. 6B). When compared among experimental groups as an average light-dependent change in surround STA peak time, there were no differences among the groups (Fig. 6C). Finally, there were no IOP-dependent effects among RGC subtypes (Fig. 6D and Supplementary Table S1; *P* = 0.92).

## Discussion

RGC responses are highly dynamic and functionally dependent on ambient light.^27^ RGCs adjust their behavior, including spatial and temporal tuning,^22^^,^^24^^,^^28^ contrast gain,^26^^,^^28^ linearity,^23^ and even polarity,^25^^,^^29^ according to the light level to which they are adapted. This capacity has important functional implications in contrast sensitivity, motion detection,^41^ and natural scene perception.^42^ Using MEA recording of individual RGCs in the bead model of IOP elevation, this manuscript clarifies several new light-dependent spatial and temporal changes in normal RGCs and the effect of IOP level on these adaptations. Interestingly, we detected several changes in RGC properties after exposure to low (low IOP) but not high (high IOP) levels of IOP elevation. This implies that some aspects of RGC physiology are extremely sensitive to IOP, and that their detection may only occur in a short window shortly after IOP elevation and prior to widespread progressive RGC injury due to higher IOP levels.

In this manuscript, we exclusively studied RGCs with both photopic and scotopic responses. Almost all such RGCs displayed some degree of center RF size alteration with a change in lighting from scotopic to photopic levels (Fig. 3A).^35^ Many had a larger center RF under scotopic light, a finding that is consistent with published works.^22^^,^^43^ However, for many other RGCs, RF center size changed in the opposite direction. Overall, the effects on the population were small, despite a wide range of RF center variation within the entire population (Fig. 3B, 3C). We believe that this is the result of functional discretion among the RGCs, for example, not all RGCs adapt to light in the same way in terms of spatial selectivity.^43^ There was no obvious difference in this RGC property because of IOP elevation or among RGC subtypes.

To expand our study beyond RF size, we analyzed the dynamics of RF spatial structure through center-surround interactions. Antagonistic surround is effectively detected by checkerboard white noise stimulation, and an empirical 1-to-3 σ versus 4-to-9 σ spatial schematic successfully separates center and surround RFs in most RGCs.^18^^,^^31^^,^^32^^,^^35^^,^^44^ We have previously demonstrated that under photopic lighting, surround strength measured by this way was negatively affected by IOP elevation.^18^ However, increased IOP may also impair RF surround on a more dynamic level. In this manuscript, we used a novel, general computational approach and introduced new metrics (transitional zone and its spatial shift) to investigate the IOP effects on spatial dynamics between center and surround RF. The transitional zone reflects the spatial extent where surround RF exerts the most strength relative to center RF. With this method, we found that transitional zone shifting was highly dynamic depending on lighting conditions. In a majority of RGCs, there was a sharpening of the central RF, manifested by a large spatial shift of the transitional zone, when the cell was exposed to brighter ambient light (Fig. 4, Table). This is in line with classical studies that identified changes in surround strength with dark adaptation^45^^,^^46^ and presumably occur through surround recruitment mechanisms in the inner retina.^22^^,^^43^ Among RGCs exposed to low levels of IOP elevation, however, we found that this process of transitional zone shift with surround recruitment occurred much less frequently (Table), which depressed the overall magnitude of the spatial shift of the transitional zone (Fig. 4). This suggests that lighting-dependent center-surround interactions, beyond global surround strength, can be altered by pathologic conditions, such as elevated IOP. Interestingly, we did not see this effect among RGCs exposed to higher levels of IOP.

RGCs also displayed robust light-dependent temporal property changes.^24^^,^^26^^,^^28^^,^^35^^,^^44^ Specifically, under scotopic light, the STA center and surround RF peak time extended significantly in all RGCs regardless of IOP elevation or ON/OFF subtype, suggesting that these are core properties of RGCs that are resistant to the effects of IOP (Figs. 5 and 6). However, the magnitude of this property varied among experimental groups. Specifically, control (from saline injected eyes) RGCs displayed a further increase of the STA center peak time extension under scotopic light, which was mitigated by elevated IOP, regardless of level. A similar procedural effect with this model has been previously reported.^18^

How can we explain the light-dependent spatial and temporal dynamics we observed? Most interestingly, we only detected changes in the overall magnitude of the spatial shift of the transitional zone in RGCs exposed to low IOP elevation. There are at least two potential explanations for this observation: (1) higher levels of IOP induce distinct changes in RGC physiology, which differ from the effects induced by low IOP elevation; and (2) IOP level affects upstream circuitry in a variable way according to IOP level to generate these RGC effects. Either interpretation is consistent with the literature: (1) the level of IOP elevation has important transcriptional and physiologic effects that are not always linear,^18^^,^^20^^,^^21^ and there is ample clinical evidence that higher IOP leads to both a higher incidence and severity of glaucoma^9^^,^^10^^,^^47^; and (2) preganglionic gap junctions are a key structural foundation for light-dependent dynamics, and horizontal components of the retinal connectome (especially amacrine cells) are exquisitely sensitive to IOP.^12^^,^^16^^,^^28^^,^^43^^,^^48^^–^^51^ With this latter explanation, our consistent findings among RGCs exposed to low but not high IOP suggest that differential preganglionic effects may occur at different IOP levels. Further study across a wider range of IOP levels and IOP exposure durations in conjunction with extensive sampling of RGCs will be required to define the IOP level–dependent mechanisms behind these observations.

RGCs exposed to low IOP elevation were also more likely to have a scotopic STA than any other experimental group. This was an unexpected and intriguing result that may represent an “irritated” state in which additional firing occurs, making the scotopic STA more detectable. Because this increased firing is not seen in the high IOP groups, this suggests either an RGC transition to a different response state with higher IOP, or a distinct reaction at high IOP. Although we are unable to record from the same set of RGCs at more than one IOP level or across multiple time points to demonstrate this directly, an RGC transition state is implied by prior physiologic and transcriptional studies.^20^^,^^21^ If present here, this suggests that we are truly studying the most subtle changes in RGCs following IOP elevation. Additional studies to elaborate on these possibilities, why RGC irritation and scotopic STA detection are diminished with increased levels of IOP elevation and additional RGC injury, and how they underlie the light-dependent adaptation properties seen earlier will be insightful.

RGCs exposed to low IOP elevations also had a markedly increased spontaneous firing rate compared with all other groups. Again, this may represent an “irritated” RGC state brought on only by low IOP elevations. Regardless, this result is different from those seen by others, which largely report a linear reduction in spontaneous firing rate with either IOP level or duration of exposure to IOP.^15^^,^^18^^,^^19^ This discrepancy can be explained by differences in inclusion criteria; this manuscript studied exclusively RGCs with both photopic and scotopic STAs, whereas published works studied RGCs with photopic STAs and did not account for the presence of a scotopic STA. RGCs that retain scotopic responses after IOP elevation may therefore behave somewhat differently from the population. Because RGCs exposed to low IOP elevation were more likely to have a scotopic STA than all other experimental groups, they could be driving the overall spontaneous firing rates of the group to higher levels, potentially leading to an overestimate of the spontaneous firing rate of the overall RGC population. This discrepancy would be consistent with the differential RGC responses to IOP seen in several previous studies, and would expand them to include IOP level-dependent susceptibilities.^15^^,^^17^^–^^20^^,^^52^^,^^53^

## Conclusions

When the results in this manuscript are integrated with the published literature, common trends about the RGC physiologic response to IOP emerge. At low levels of IOP elevation, sensitive effects on RGC light-dependent adaptation and RGC spontaneous firing rates occur (this manuscript). However, at higher levels of IOP elevation adaptation impairment and RGC irritation appear to be replaced by other forms of RGC dysfunction, such as changes in the light-evoked response and abnormal contrast sensitivity.^16^^–^^18^^,^^20^ Finally, at even higher levels of IOP elevation, still other forms of RGC dysfunction such as reduced center RF size and widespread RGC injury highlighted initially by dendritic retraction occur.^15^^,^^19^^,^^52^^,^^53^ Thus collectively, there is compelling evidence that different levels of IOP elevation exhibit different forms of retinal dysfunction. It is therefore possible that distinct properties of IOP level–associated retinal dysfunction can be used to identify stages of disease in human glaucoma patients. Indeed, the abnormal light-dependent adaptation of RGCs to low IOP elevation described in this manuscript may represent an opportunity to identify glaucoma patients at the earliest stages, long before significant permanent injury from higher levels of IOP occurs.

## Supplementary Material

Supplement 1
