# Supplementary material for: Intraocular Pressure Elevation Compromises Retinal Ganglion Cell Light Adaptation
Source: Invest Ophthalmol Vis Sci. 2020 Oct 16;61(12):15. doi: 10.1167/iovs.61.12.15 (PMC7571289; doi:10.1167/iovs.61.12.15)
Supplement: Supplement 1 [file iovs-61-12-15_s001.pdf]

Supplemental Table and Figure for:

Intraocular Pressure Elevation Compromises Retinal Ganglion Cell Light Adaptation

Xiaofeng Tao[1], Jasdeep Sabharwal[1,2], Samuel M. Wu[1,2], and Benjamin J. Frankfort[1,2]
[1] Department of Ophthalmology, Baylor College of Medicine and [2] Department of Neuroscience, Baylor College of Medicine, Houston, Texas 77030

Supplemental Table 1. Comparisons of RGC activity and STA properties

Source	Sum of Squares	df	Mean Square	F	p
Fig. 2C: mean spontaneous firing rate (Hz)					
Subtype	58.78	2	29.39	4.99	0.0072
IOP	13.29	3	4.43	0.75	0.5214
Subtype*IOP	26.19	6	4.36	0.74	0.6165
Error	2590.19	440	5.89		
Total	2758.32	451			
Fig. 3D: mean RF size (μm)					
Subtype	2745.17	2	1372.58	5.78	0.0033
IOP	774.15	3	258.05	1.09	0.3543
Subtype*IOP	1632.42	6	272.07	1.15	0.3345
Error	104922.58	442	237.38		
Total	112223.23	453			
Fig. 4E: mean transitional zone shift (σ)					
Subtype	6.84	2	3.42	0.71	0.4905
IOP	46.70	3	15.57	3.25	0.0219
Subtype*IOP	22.54	6	3.76	0.78	0.5835
Error	2120.08	442	4.80		
Total	2214.69	453			
Fig. 5D: mean center peak time (ms)					
Subtype	4345.92	2	2172.96	2.66	0.0714
IOP	38199.17	3	12733.06	15.56	0.0000
Subtype*IOP	7052.39	6	1175.40	1.44	0.1989
Error	361620.20	442	818.15		
Total	524812.33	453			
Fig. 6D: mean surround peak time (ms)					
Subtype	224846.47	2	112423.23	1.74	0.1799
IOP	13245.34	3	4415.11	0.07	0.9767
Subtype*IOP	129612.04	6	21602.01	0.33	0.9180
Error	8409026.08	130	64684.82		
Total	8842836.01	141			

df: degree of freedom
Subtype: RGC subtypes, including ON, OFF, and ON-OFF
IOP: eye conditions, including Normal, Control, Low IOP, and High IOP

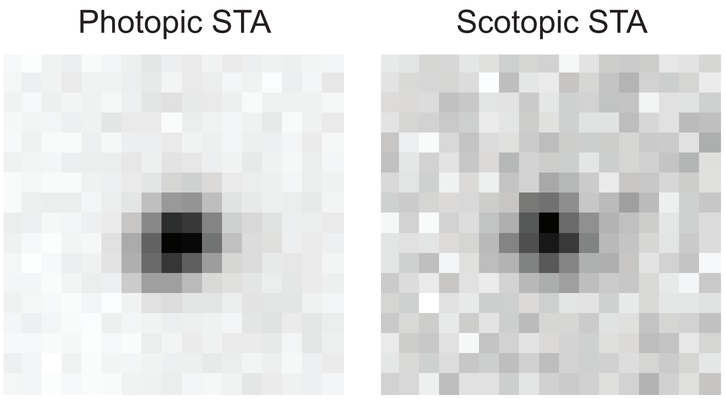

Supplemental Figure 1. Photopic and scotopic STAs of a representative RGC. This OFF type RGC was recorded from a normal (uninjected) eye. The size of the center photopic RF is 71.1 μm and the size of the center scotopic RF is 73.6 μm . The area for each panel is 850 x 850 μm.
